# Genes, animal models and the current understanding of psychiatric disease 

**DOI:** 10.1186/1741-7007-9-78

**Published:** 2011-11-11

**Authors:** Penelope Austin

## 

Psychiatric disorders affect approximately 1 in 4 people. Two articles published today in BMC Biology [1,2] discuss prospects for an improved understanding of their biology and treatment, informed by recent and ongoing advances in defining the genetic architecture that underlies them. 

Although neuropsychiatric diseases show substantial heritability, their inheritance is not simple. As for other common diseases that do not show simple Mendelian inheritance, the prevailing hypothesis of the last half-century has been that many common alleles, each of modest effect, combine with environmental factors to cause disease. For neuropsychiatric disorders, however, this thinking has been challenged by recent findings of an increasing number of rare mutations that have a large effect on disease susceptibility. 

In their review article [1], Kevin Mitchell, Josh Huang, Bita Moghaddam and Akira Sawa lay out a framework for studying the effects of these rare variants in animals, explaining why they see this as the best way to model psychiatric disease.  Previously, animal models were developed on the basis of behavioral assays that appeared to represent some aspect of psychopathology, and viewed as valid when the behaviors in question were modified by medication used to treat the disease. Such models have yielded few new drugs, whereas modeling causal genetic variants in animals offers the possibility of identifying disease pathology at multiple levels and developmental timepoints, and the hope of new targets of direct relevance for therapeutic intervention in humans.

In a separate article [2]  Aiden Corvin, a psychiatrist and member of the Psychiatric Genetics Consortium responsible for recent genome-wide association studies (GWAS) on psychiatric disease, considers the implications of recent genetic findings for schizophrenia patients. Currently, schizophrenia is diagnosed on the basis of combinations of symptoms, no one of which is on its own specifically diagnostic of schizophrenia; and the few treatments available are only partially effective, and only in a subset of patients. Corvin looks to a future in which diagnosis will be guided by molecular etiology, and patients may be treated with drugs known to be effective for the particular genetic subtype of disease. 

One of the most striking findings of the genetic studies is that individual rare mutations of large effect often confer susceptibility to more than one disorder, for example both schizophrenia and autism. Overlap in susceptibility is also seen in the GWAS that have to date identified 10 common variants with small effects on disease risk, though here the major overlap is seen with bipolar disorder. These findings are consistent with epidemiological studies that indicate shared risk for different disorders, and families in which a spectrum of disorders is observed. The strong association of different disorders with the same mutation suggests they can be distinct phenotypic endpoints that can arise from a common genetic origin, rather than a polygenic effect, a possibility that can be explored using the modeling approach outlined by Mitchell and colleagues. Corvin concludes from this accumulating evidence that schizophrenia will not continue to be viewed as a single disease.

In one respect there is a notable difference in the stance taken by the two articles.  Mitchell and coauthors espouse the proposition, gaining ground of late [3,4], that the disease-associated rare variants reported so far (mostly short deletions or duplications) represent only the tip of the iceberg, and that as sequencing studies uncover more functionally relevant mutations, most cases of neuropsychiatric disease will be found to be attributable, in large part, to single rare mutations of large effect. Corvin on the other hand takes the view that with only 5% of cases so far explained by rare mutations, it is too early to judge to what extent they account for the heritability of the disease, and that the combined effect of common alleles, together with environmental factors, may still explain a considerable fraction of cases [5]. These two different outlooks reflect a lively and ongoing debate on the genetic architecture of neuropsychiatric disease, the value of proceeding to yet bigger GWAS studies in an effort to identify more common risk loci, and to what extent common risk variants may actually be tagging linked rare mutations responsible for the association with disease [6,7]. The expectation is that answers will come with the increasing number of patient genomes or exomes being sequenced, alongside those of family members – though this is not without difficulties, as a typical person is estimated to carry about 150 rare coding variants affecting about 1% of his or her genes, making it a challenge to identify those with a causal role in disease.   

Wherever the final balance lies, there is agreement that the rare mutations of large effect provide a much-needed entry point to get at the underlying biology of these disorders. The hope is that despite the heterogeneity of primary genetic causes, systematic analyses in animal models, using the sophisticated neurogenetic tools now available, will identify a convergence of pathogenic mechanisms that can be targeted therapeutically.
